# Latest Progresses in Allergic Diseases Biomarkers: Asthma and Atopic Dermatitis

**DOI:** 10.3389/fphar.2021.747364

**Published:** 2021-09-30

**Authors:** Pier Giorgio Puzzovio, Francesca Levi-Schaffer

**Affiliations:** Pharmacology and Experimental Therapeutics Unit, Faculty of Medicine, School of Pharmacy, Institute for Drug Research, The Hebrew University of Jerusalem, Jerusalem, Israel

**Keywords:** allergic inflammation, asthma, atopic dermatitis, biomarkers, eosinophils, neutrophils

## Abstract

In the last years, the understanding of the pathologic mechanisms of asthma and atopic dermatitis, both characterized by allergic inflammation, has greatly improved. However, it is evident that both diseases present with high heterogeneity, which complicates the diagnosis and the therapeutic approach of the patients. Moreover, some of the currently available strategies to treat asthma and atopic dermatitis are still mostly controlling the symptoms, but not to lead towards full healing, thus having these two diseases labelled as unmet clinical needs by WHO. Therefore, the “one-size-fits-all” strategy is outdated for asthma and atopic dermatitis, and there is the need of better methods to clearly diagnose the disease and tailor the therapy according to the specific symptomatology. In this regard, the use of biomarkers has been advanced in order to characterize both diseases according to their clinical signs and to facilitate the subsequent treatment. Despite the advancements made in this regard, there is still need for better and more sensitive biomarkers and for less invasive sampling methodologies, with the aim to diagnose specifically each manifestation of asthma and atopic dermatitis and to provide the best treatment with the least suffering for the patients.

## Introduction

In recent years, advancements of medical research in the field of allergic diseases have led to a better understanding that pathologies characterized by allergic inflammation (AI) are heterogeneous and present with a high degree of variability among patients (Roth and Stolz, 2019; Bakker et al., 2020). Moreover, most of the currently available treatments are still only able to ease the symptomatology/symptoms, to decrease inflammation and some of them to partially prevent exacerbations and perhaps to modify the natural course of the disease. However, even the newest biologic-based drugs are not able to cure it. This might be due to the concomitant presence of an atopic condition together with the inability of the atopic individual to fully resolve the inflammation. Thus, allergic diseases and especially severe asthma and atopic dermatitis have been labelled by the WHO as unmet clinical needs (Breiteneder et al., 2019). Given the advances in research, and in light of the concept of personalized medicine, the necessity of finding novel and more accurate biomarkers for allergic diseases has been raised. A biomarker (or biological marker) is defined as a “characteristic that is objectively measured and evaluated as an indicator of normal biologic processes, pathogenic processes, or pharmacologic responses to a therapeutic intervention” according to the National Institute of Health. Consequently, biomarkers evaluation should help in the diagnosis of the disease as well as in predicting its outcomes and the effects of the prescribed therapy (Narendra et al., 2019).

## Biomarkers for Asthma

Asthma is a lung disease characterized by sometimes irreversible bronchoconstriction, airway hyperresponsiveness, chronic inflammation, mucus hypersecretion and tissue remodeling (Lambrecht and Hammad, 2015). In the last 40 years its prevalence and morbidity have increased, with approximately 300 million individuals affected worldwide and a total of $80 billion dollars yearly expenses (Narendra et al., 2019). Over the years, several attempts have been made to better characterize the etiopathology of the disease, but still there is no effective therapy for all the spectrum of asthma forms, especially for the severe ones. Indeed in mild/moderate cases, asthma symptoms and underlying inflammation can mostly be controlled with the use of inhaled *β*-adrenergic agonists, muscarinic antagonists and glucocorticosteroids and other available anti-inflammatory drugs (Lambrecht and Hammad, 2015; Koarai and Ichinose, 2018). More recently, the use of monoclonal antibodies such as anti-IgE, anti-IL-5, anti-IL-5R, and anti-IL-4/IL-13R*α* has been demonstrated to be able to control asthma pathogenesis and hence symptoms, but not to resolve the disease. Thus, as mentioned above, asthma remains an unmet clinical need.

### Asthma Endotypes and Phenotypes

In recent years, asthma has been defined as a disease characterized by heterogeneous features, which include the type of inflammation, presentation of the symptoms, response to treatments and long-term consequences for the patients. Therefore, asthma is better characterized by defining endotypes and phenotypes (Narendra et al., 2019; Roth and Stolz, 2019). Asthma endotypes encompass the pathologic mechanisms underlying the disease, while phenotypes include its clinical manifestations (Kuruvilla et al., 2019). Endotypes classification allows asthma to be divided into type 2 and non-type 2 asthma, although there is evidence of different subtypes linked to the inflammasome and skin structural components, and mixed 2, 1, 17 subtypes (Agache and Akdis, 2019). Asthma phenotypes include patients’ features (age, gender, ethnicity, etc.), morphophysiological characteristics of the airways, response to therapies and clinical outcomes (Agache and Akdis, 2019). The emergence of the concept of asthma endotypes and phenotypes has prompted the need of a better understanding of the disease characteristics, in order to have a more personalized therapeutic approach and to predict the outcomes of the treatment. Therefore, the use of biomarkers to define the specific presentations of asthma has been advanced, each of them with their pros and cons.

### Biomarkers for Asthma

In order to be eligible for asthma, a biomarker should be “superior, actionable, valuable, economical, and clinically deployable” (Diamant et al., 2019). Biomarkers for asthma are mainly divided into biomarkers for type 2 and non-type 2 asthma and might be sampled from different sources, with several advantages and disadvantages (Diamant et al., 2019; Narendra et al., 2019).

#### Biomarkers for Type 2 Asthma

##### Eosinophils

One of the main biomarkers for type 2 asthma is the eosinophil (Eos) numbers, which are preferably analyzed in the blood and in the sputum of asthmatic patients due to the lower invasiveness of these methods, although with lower reproducibility and higher technical complexity (Diamant et al., 2019). Increased Eos counts in the blood (>400 cells/μl) were associated with higher prevalence of exacerbations and lower possibilities to control the disease (D. B. Price et al., 2015). However, Eos blood counts for asthma are not fully reliable, since blood eosinophilia might be due to other T2 inflammation-inducing conditions, such as parasitic infections or some autoimmune diseases (Narendra et al., 2019). Nevertheless, blood eosinophilia is a good marker to follow the T2 inflammation after treatment with anti-IL-5 (mepolizumab and reslizumab), anti-IL-5R*α* (benralizumab) and anti-IL-4 (dupilumab) biologics (Castro et al., 2011; Wenzel et al., 2013; Ortega et al., 2014; FitzGerald et al., 2016), as high blood Eos count is considered a good predictive value for the response to the aforementioned biologics (Cevhertas et al., 2020). Sputum Eos is believed to be the most accurate method to assess eosinophilic asthma (a value higher than 2% Eos is considered indicative of airway inflammation) (Westerhof et al., 2015; Walsh et al., 2016). A more refined technique to distinguish between type 2 and non-type 2 asthma involves analysis of the sputum mRNA levels of Th2 cytokines (Seys et al., 2017). Sputum Eos have been historically employed to follow the outcomes of corticosteroid treatment, since lower eosinophilia correlated with reduced exacerbations and hospitalizations after inhaled corticosteroids (ICS) administration (Morrow Brown, 1958; Green et al., 2002; Fitzpatrick et al., 2016). Another technique employed to analyze Eos in asthmatic patients is bronchoscopy, which is performed via biopsies, bronchoalveolar lavage or bronchial brushing. However, the higher invasiveness and complexity of these techniques limits their application (Diamant et al., 2019). Another possibility involves measurement of Eos granule proteins, such as Eos peroxidase, Eos cationic protein and Eos-derived neurotoxins, which have been found to decrease after administration of anti-Eos biologics (Narendra et al., 2019).

##### Fraction of Exhaled Nitric Oxide

FeNO measurement is an indirect indication of airway inflammation, since it correlates with Eos counts in the lungs (Fajt and Wenzel, 2015). Indeed, FeNO is linked to eosinophilia, according to the American Thoracic Society recommendations, (>50 ppb and >30 ppb for adults and children, respectively) (Dweik et al., 2011). FeNO is directly measurable in the exhaled breath of patients. The sampling method is easy and non-invasive, and the results are reproducible, prompting its use also for pediatric asthma (Neerincx et al., 2017). However, the results might be influenced by several factors, such as age, smoking habits, drug use, which should be taken into consideration when performing the measurement (Buchvald et al., 2005; Borrill et al., 2006). Even though it was found that FeNO levels decrease in response to inhaled corticosteroids (ICS) and dupilumab treatment (D. Price et al., 2013; Wenzel et al., 2013), the ERS/ATS guidelines suggest to avoid the use of FeNO as a predictive marker for therapy in severe asthma (Chung et al., 2014).

##### Serum IgE

Total serum IgE were found to be increased in allergic asthmatic adults and children, and their levels increased with disease severity (Burrows et al., 1995). Moreover, high serum IgE levels are indicative of sensitization to an allergen, which makes patients eligible for treatment with the monoclonal anti-IgE antibody omalizumab. However, it was demonstrated that, despite reducing serum IgE, total IgE levels do not change significantly after omalizumab administration, due to the fact that omalizumab binds to free IgE and forms complexes with them, thus increasing the total IgE levels (Humbert et al., 2014). However, its use was found to reduce the incidence of asthma exacerbations (Humbert et al., 2014). Therefore, this marker does not allow to predict the response to treatments in asthmatic patients, and must be analyzed together with other biomarkers.

##### Periostin

Periostin is produced and released by epithelial cells after stimulation with IL-13, a cytokine indicative of Th2 inflammation (Izuhara et al., 2016). Periostin’s effectiveness as a biomarker was shown to be higher than Eos, FeNO and serum IgE in patients with uncontrolled severe asthma and ICS treatment (Jia et al., 2012). Moreover, patients with high periostin levels were found to show reduced asthma exacerbations after treatment with lebrikizumab (anti-IL-13), demonstrating the prognostic value of serum periostin concentrations (Hanania et al., 2015). However, its levels fluctuate with age (Narendra et al., 2019) and can change also during other inflammatory processes such as atopic dermatitis, eosinophilic esophagitis and cancer, requiring its use in addition to other markers (Izuhara et al., 2016). In addition, the existence of different splicing variants of periostin complicates the measurement of this biomarker.

#### Biomarkers for Non-type 2 Asthma

##### Neutrophils

Higher neutrophil counts in the sputum and in the blood have been associated with severe forms of asthma (Moore et al., 2014; Ricciardolo et al., 2018). However, there is no clear definition of neutrophilic asthma, since different threshold values for neutrophil levels were reported in the literature (Simpson et al., 2006; Moore et al., 2014). Moreover, airway neutrophilia was also found to be induced by use of oral corticosteroids (Alam et al., 2017), but also by other conditions, such as obesity, smoking habits, gastroesophageal reflux or lung infections (Ray and Kolls, 2017). This complicates the diagnosis of neutrophilic asthma, since some of its features are mistakenly ascribed to other diseases, such as chronic obstructive pulmonary disease, or effects of smoking (Gibson and Foster, 2019). Generally, a value between 61 and 76% is considered indicative of neutrophilic airway inflammation, although no real consensus exists about these threshold values (F. Schleich et al., 2016).

##### Serum Cytokines

The main markers associated with neutrophilic asthma are IL-17, IL-8, and TNF*α*, since their levels in the serum were found increased in asthmatic patients with neutrophilia (Diamant et al., 2019). A positive correlation was found between neutrophil numbers and IL-17 mRNA levels in the sputum (Bullens et al., 2006), and it was found that bronchial epithelial cells released IL-8 after stimulation with IL-17, contributing indirectly to IL-8-induced neutrophils recruitment (Lindén, 2001). However, this marker did not show any therapeutical predictive value since treatment with an anti-IL-17R biologic, brodalumab, did not prove to be effective in severe asthma (Busse et al., 2013). Tightly linked to airway neutrophilia is the cytokine IL-8, since it is known to be a chemoattractant for neutrophils and it has been found in high levels in BAL and sputum uncontrolled asthmatic patients (Gao et al., 2017). Moreover, expression of IL-8 receptors CXCR1-2 was found to be increased in the sputum of neutrophilic asthmatic patients (Wood et al., 2012). In this regard, the use of CXCR2 antagonists was shown to decrease neutrophils count in the sputum and to reduce mild exacerbations (Nair et al., 2012), further bolstering the connection between neutrophils and asthma. Other cytokines involved in neutrophilic asthma include IL-4, which might indirectly induce neutrophil migration and activation by regulating the expression levels of IL-8, TNF-α, and IL-1β in correlation with the severity of the disease (Lavoie-Lamoureux et al., 2010). Therefore, IL-8 and IL-4 might be helpful in distinguishing between airway neutrophilia due to asthma or other pathological conditions. TNFα levels were found to be increased in the sputum of neutrophilic asthmatics (Simpson et al., 2007) and to positively correlate with both NO and neutrophil numbers in severe asthmatic patients (Silvestri et al., 2006). Interestingly, treatment with the anti-TNFα etanercept improves airway hyperresponsiveness and quality of life in refractory asthma, and this improvement positively correlated with etanercept-induced reduction of membrane-bound TNFα expression (Brightling et al., 2008). Thus, TNFα might be used also as a predictive biomarker for etanercept therapy. Other markers are related to neutrophil activation and include sputum myeloperoxidase and elastase, which were detected in high levels in neutrophilic asthma (F. Schleich et al., 2016).

#### Novel Biomarkers

##### Airway Remodeling

One of the main features of asthma is the airway remodeling, involving airways obstruction, mucus hypersecretion, angiogenesis, and excessive fibrosis (Bergeron et al., 2009). Unfortunately, at the moment there is no clear-cut marker for airway remodeling, and the preferred method to analyze remodeling in the airways is the bronchial biopsy, which is highly invasive and risky. Furthermore, the great variability in the tissue raises the necessity of having more than one sample (Diamant et al., 2019). Some less-invasive markers include sputum matrix metalloproteinase 2 (MMP-2), fibroblast growth factor 2 (FGF-2) and galectin-3, the latter being predictive of omalizumab effects on airway remodeling (Mauri et al., 2014; Elkolaly and Ali, 2018; Sivakoti et al., 2018; Tan et al., 2020). Other airways remodeling markers include CCL16, released by bronchiolar exocrine cells, which is usually measured in the sputum and compared to IL-8 levels (F. Schleich et al., 2016).

##### Volatile Organic Compounds

VOCs are a collection of molecules derived from the metabolism of different endogenous or exogenous compounds. These molecules were found to differ between eosinophilic and neutrophilic asthma. For example, hexane and 2-hexanone were found to be characteristic of eosinophilic asthma, with similar accuracy to FeNO and blood Eos (F. N. Schleich et al., 2019). On the other hand, the molecules found in high concentrations in neutrophilic asthma are nonanal, 1-propanol and hexane (F. N. Schleich et al., 2019).

##### Specialized Pro-resolving Mediators

SPMs are a class of lipid molecules encompassing different families with their biosynthetic pathways and receptors, all of them implicated in the resolution of inflammation (Fullerton and Gilroy, 2016). SPMs levels might be analyzed in a wide number of biological materials, such as blood, sputum, bronchoalveolar lavage (BAL), exhaled breath condensates, as well as in urine, breast milk and tears, in their bioactive concentration (pg/ml) (Serhan, 2014). Notably, SPMs pathways are reduced in severe asthma patients. Specifically, lower lipoxin A4 (LXA4) levels were found in the BAL of severe asthmatic patients (Planaguma et al., 2008), correlating with decreased lung functions. Moreover, severe asthma patients have been found to present with reduced docosahexaenoic acid (DHA) concentrations in the airways’ mucosa, hinting that production of protectin D1 and D-resolvins might be impaired as well (Freedman et al., 2004). It was also found that in severe asthma the expression of ALX/FPR2, the receptor binding resolvin D1 and LXA4, is reduced on peripheral blood Eos and neutrophils and increased on BAL macrophages and neutrophils and peripheral blood natural killer (NK) cells (Planaguma et al., 2008; Barnig et al., 2013; Ricklefs et al., 2017). This evidence would indicate that SPMs and their pathways might be a good candidate for detecting severe asthma and the consequent defective resolution.

##### A Potential New Marker: sCD48

CD48 is an activating receptor expressed on immune cells which exists in a membrane-bound form and a soluble one (sCD48) (Smith et al., 1997). CD48 on mast cells was found to interact with CD244 on Eos, initiating a cross-talk with marked pro-inflammatory outcomes in AI, the Allergic Effector Unit (Elishmereni et al., 2013). The expression of CD48 was found to be increased in Eos from peripheral blood and nasal polyps of mild asthmatic patients (Munitz et al., 2006a) and on NK cells, B-cells and T-cells of severe asthmatic patients (Gangwar et al., 2017). The levels of CD48 soluble form, sCD48, were higher in the serum of mild asthmatic patients and reduced in moderate and severe asthma (Gangwar et al., 2017). Interestingly, sCD48 levels in asthmatic patients did not correlate with Th2 inflammation markers, and this prompted the hypothesis that its expression might be linked to a broader role in inflammatory processes rather than specific AI (Breuer et al., 2018). Therefore, CD48 might be a good candidate as a biomarker for different degrees of asthma severity.

## Biomarkers for Atopic Dermatitis

Atopic dermatitis (AD) is among the most common inflammatory skin diseases (Nomura et al., 2020). The lack of a proper therapeutic strategy against AD has rendered this disease a significant socioeconomic burden worldwide, with higher prevalence amongst children (Barbarot et al., 2018). In AD there is increasing evidence pointing to a high degree of heterogeneity in clinical manifestations and molecular characteristics, advancing the concepts of endotypes/subtypes also for AD (Bakker et al., 2020). As with asthma, treatments for AD are moving towards the concept of personalized medicine, mostly due to the heterogeneity of the disease. This is most important, since AD is still dealt with the “one-size-fits-all” approach, which greatly limits the effectiveness of the treatment (Bieber et al., 2017).

### AD Subtypes

Over the years, the characterization of AD has been significantly elucidated thereby shedding light on the complexity of this disease. This led to the classification of AD manifestations into different subtypes, namely age-related features, severity of the disease, age of onset and ethnicity according to skin condition, presence of lesions, and underlying inflammation (Bieber et al., 2017).

This classification is mainly based on the severity and the extension of the lesions and of the skin conditions, and it employs diagnostic scores such as the SCORAD or Eczema Area and Severity Index (Bieber et al., 2017). However, the underlying inflammatory response in the patients is also taken into consideration and used to define the disease characteristics. The immunological profile of AD patients shows a marked Th2 inflammation in all the subtypes, as shown by frequencies of IL-13^+^ and IL-4^+^ T-cells (Esaki et al., 2016b), while Th22 inflammation increased from infancy to adulthood, as shown by high levels of IL-22 in adult AD in comparison to infancy AD (Czarnowicki et al., 2020). In childhood AD, Th17 and Th9 responses were found, as demonstrated by the higher levels of cytokines such as IL-17A, IL-19 and IL-9, respectively (Esaki et al., 2016a). This immunological response might change according to the ethnicity of the patients. Asian patients present with increased Th17/Th22 inflammation, shown by the increased skin thickness and Th17/22 markers expression in skin and blood, with “psoriasis-like” manifestations (Noda et al., 2015). On the other hand, African Americans displayed increased Th22 response and skin barrier defects, while Caucasian patients showed induction of Th22, Th17 and Th1 inflammation, with reduced production of skin barrier proteins (Nomura et al., 2020). In all ethnicities the Th2 response was always present.

This evidence shows that AD heterogeneity comprises many factors, that complicate the diagnosis and the consequent treatment. Therefore, as for asthma, also in AD biomarkers have been proposed to facilitate the definition of the disease severity.

### AD Biomarkers

In contrast to what was seen in asthma, there is a general lack of suitable biomarkers for AD, mostly due to the difficulties inherent to sample retrieval. Indeed, most of the existing knowledge regarding AD biomarkers is obtained from studies performed on skin biopsies, which is an invasive and potentially dangerous method especially for infants. Therefore, new sampling methods are being used, such as skin tape-stripping, for following both the disease and the treatments (Castelo-Soccio, 2019; Guttman-Yassky et al., 2019). Another source of samples for AD biomarkers analysis is the serum of the patients (Ungar et al., 2017). Other sampling methods, less invasive, are dried blood spots (DBS), consisting in droplets of blood collected via a capillary and absorbed on a cellulose layer. DBS are then eluted via an adequate buffer and processed for biomarkers analysis. This technique is minimally painful and easy to process (J. L. Thijs et al., 2019). Another less invasive source of samples is saliva, mainly due to the possibility of blood biomarkers diffusing into the salivary glands (Thijs J. et al., 2015). Biomarkers for AD in the skin are generally measured via their mRNA levels, while serum biomarkers can be analyzed also via ELISA (Ungar et al., 2017; Guttman-Yassky et al., 2019). Identification of potential patients before symptoms appearance involves evaluation of the skin barrier functionality, via epidermal water loss and expression of FLG1-2, encoding for the proteins filaggrin 1 and 2, or other structural skin proteins (Margolis et al., 2014; Bager et al., 2016). The stratification of patients is done also according to the underlying immunological response. AD is mainly a Th2-driven disease, characterized by expression of IL-4, IL-5 and IL-13, and the levels of these cytokines can also predict the outcomes of anti-IL-4, anti-IL-5 and anti-IL-13 biologics therapies (Bakker et al., 2020). Where the phenotype is more Th17-dominant, the levels of IL-17 are evaluated, and might be a good marker for following anti-IL-17-directed approaches (Bieber et al., 2017). In patients showing upregulation of the Th22 response, IL-22 is the most evaluated marker for disease severity, and its levels were also shown to be predictive of fezakinumab (anti-IL-22) administration outcomes (Brunner et al., 2019). Other markers for AD severity include ECP, TSLP, *β*-defensin 1, eotaxin, RANTES, CCL17-22-27, the latter allowing T-cells homing to the skin (Bieber et al., 2017; Bakker et al., 2020). Among these markers, CCL17 was the one showing the highest correlation with AD severity, although its levels are generally variable with AD heterogeneity and underlying inflammatory pathways (Landheer et al., 2014; Thijs J. L. et al., 2015). Sensitization to the allergen is measured via total and specific IgE, however the IgE profile is subject to great variation among the population. Therefore, it would be more useful to examine the ratio between specific and total IgE (Bieber et al., 2017).

#### New Potential Biomarkers

##### Adipokines

In a recent study, the levels of serum adipokines were evaluated and related to the disease features. Two adipokines, adiponectin and resistin, showed lower levels in AD patients and an inverse proportionality trend with the severity of the disease. On the other hand, leptin levels were increased in AD patients, but did not correlate with disease severity. No correlation between adipokines’ levels and patients’ characteristics (age, gender, BMI) was found (Jaworek et al., 2020). Thus, although requiring more studies, adipokines might be a new interesting and more specific set of biomarkers linked to AD severity.

##### CD300a

CD300a is an inhibitory receptor expressed on the surface of several immune cells, and its role in downregulating AI has been extensively demonstrated (Bachelet et al., 2005; Bachelet et al., 2006; Munitz et al., 2006b). It was recently published that total CD300a expression is increased in lesional AD skin and specifically on Eos, and that its expression positively correlated with hypoxic conditions and angiogenesis in AD skin (Karra et al., 2019). Moreover, CD300a expression was significantly increased on B-cells from AD patients and decreased on circulating NK cells (Karra et al., 2019). Interestingly, CD300a expression was not increased in non-lesional AD skin [(Karra et al., 2019) supporting information)], hinting that this receptor might be a marker for severe forms of the disease.

##### CD48

CD48 surface levels were found to be significantly decreased in peripheral blood from mild/moderate/severe AD patients, and on Eos, neutrophils, monocytes, basophils, NK cells, T- and B-cells (Minai-Fleminger et al., 2014). However, its expression was significantly increased on Eos in biopsies from lesional AD skin (Minai-Fleminger et al., 2014). It was hypothesized that this differential CD48 expression might be the result of CD48 sensitivity to local stimuli rather than systemic ones (Minai-Fleminger et al., 2014). Thus, CD48 might provide information regarding local inflammation in AD lesional skin.

##### Skin Microbiome

The role of secondary infections in AD, especially from bacteria such as *Staphylococcus aureus*, normally residing on the skin, is well characterized (Weidinger and Novak, 2016). A recent study has shown that AD skin presents with dysbiosis in comparison to healthy controls, with prevalence of *Staphylococcus aureus* and reduction in anaerobic bacteria species, correlating with disease severity (Fyhrquist et al., 2019). In another study, the skin microbiome composition was employed to divide patients in “dermotypes”, each one with distinct bacterial genera prevalence and metabolic profiles (Tay et al., 2020). Among the dermotypes, the “B” one presented with higher Th2-specific mediators, worsened symptomatology and increased possibility to develop other atopic diseases (Tay et al., 2020). This evidence adds another level of characterization of AD, which might aid in stratification of the patients and evaluation of biomarkers.

## Concluding Remarks

Since the emergence of the concept of personalized medicine, it has become clear that the “one-size-fits-all” approach for allergic diseases is not adequate to treat the high heterogeneity of patients. Thus, the search for biomarkers for predicting the occurrence and the outcomes of the disease was prompted. Despite the vast amount of research conducted, there is still a need for less invasive sampling techniques and more sensitive markers.

New techniques include the use of -omics technology, such as transcriptomics, proteomics and metabolomics, to create a detailed profile of the asthma features. One of the latest applications is the metabolic profiling of the breath of asthma patients before and after ICS treatment (Ferraro et al., 2020). Moreover, the aim of the ongoing SMART clinical trial (NCT04194814), started in 2019, is to test new non-invasive methods to evaluate biomarkers for skin structure and function variations.

In conclusion, biomarkers for asthma and AD provide useful tools in the diagnosis of the disease and the prediction of the symptoms’ occurrence and therapeutical responses/outcomes. Novel methodologies for both sampling and analysis are now being evaluated. In time, this might result in better analytic strategies that would benefit both the patient and the clinician in terms of non-invasiveness, reliability and specificity of the marker, in order to design the best therapeutical approach for each patient.
